# Multiresponse Optimization of Process Parameters in Turning of GFRP Using TOPSIS Method

**DOI:** 10.1155/2014/905828

**Published:** 2014-10-29

**Authors:** Arun Kumar Parida, Bharat Chandra Routara

**Affiliations:** School of Mechanical Engineering, KIIT University, Bhubaneswar, India

## Abstract

Taguchi's design of experiment is utilized to optimize the process parameters in turning operation with dry environment. Three parameters, cutting speed (*v*), feed (*f*), and depth of cut (*d*), with three different levels are taken for the responses like material removal rate (MRR) and surface roughness (*R*_*a*_). The machining is conducted with Taguchi L_9_ orthogonal array, and based on the *S*/*N* analysis, the optimal process parameters for surface roughness and MRR are calculated separately. Considering the larger-the-better approach, optimal process parameters for material removal rate are cutting speed at level 3, feed at level 2, and depth of cut at level 3, that is, *v*_3_-*f*_2_-*d*_3_. Similarly for surface roughness, considering smaller-the-better approach, the optimal process parameters are cutting speed at level 1, feed at level 1, and depth of cut at level 3, that is, *v*_1_-*f*_1_-*d*_3_. Results of the main effects plot indicate that depth of cut is the most influencing parameter for MRR but cutting speed is the most influencing parameter for surface roughness and feed is found to be the least influencing parameter for both the responses. The confirmation test is conducted for both MRR and surface roughness separately. Finally, an attempt has been made to optimize the multiresponses using technique for order preference by similarity to ideal solution (TOPSIS) with Taguchi approach.

## 1. Introduction

Composite materials are replacing the traditional materials because of their light weight, high modulus, high specific strength, and low thermal expansion. It is also an economically alternative to stainless steel. In recent years glass fiber reinforced polymer (GFRP) composites are extensively used in various fields such as aircraft, gas industries, oil industries, processing industries, and defense. GFRP composite components are generally fabricated by filament winding and hand lay-up process. After fabrication of components they further require machining for dimensional control in easy assembling. During machining of GFRP composites, high dimensional accuracy and better surface finish are the necessary qualities on the machined surface. Many of the machining results show that it is too difficult to minimize the surface roughness but it is controlled. According to Hussain et al. [1], the surface roughness has been controlled with moderate cutting speed, low feed and moderate depth of cut while machining of GFRP with carbide K-20 tool. Feed is the most dominant parameter which affects the surface roughness followed by orientation angle and speed. Depth of cut shows a minimal effect in comparison to other parameters. Palanikumar [2] used Taguchi and response surface methodology in minimizing the surface roughness using PCD tool. The experimental response showed that the significant parameter is feed followed by velocity and the depth of cut has the minimum effect on roughness in comparison to other cutting parameters. Davim et al. [3] investigated the influence of cutting parameters on the surface roughness using cemented carbide and polycrystalline diamond cutting tools. The investigation reports that the feed rate is the cutting parameter that has the highest physical as well as statistical influence on the surface roughness. Ramulu et al. [4] carried out study on machining of polymer composites. They concluded that higher cutting speed gives better surface finish. But according to Takeyama and Lijima [5], higher cutting speed produces more damages on the machined surface. This enables higher cutting temperature, which results in local softening of work material. Wang and Zhang [6] investigated the machinability of epoxy composites reinforced by unidirectional carbon fiber materials when subjected to orthogonal cutting and found that the subsurface damage and its mechanisms of a machined component are greatly influenced by fiber orientation. Davim and Mata [7] studied the machinability in turning processes of FRPs using polycrystalline diamond cutting tools. In their study, controlled machining experiments are performed with cutting parameters prefixed in the workpiece. A statistical technique, using orthogonal arrays and analysis of variance, is employed to investigate the influence of cutting parameters on specific cutting pressure and surface roughness. Davim and Mata [8] also presented an optimization study of surface roughness in turning FRP tubes manufacturing by filament winding and hand lay-up using polycrystalline diamond cutting tools. Palanikumar et al. [9] investigated machining of GFRP in an attempt to assess the influence of machining parameters on the machining of GFRP. A procedure was developed to assess and optimize the chosen factors to attain minimum surface roughness. It was found that, for achieving good surface finish on the GFRP workpiece, high cutting speed and high depth of cut are required. Depth of cut shows minimum effect on the surface roughness compared to other parameters.

So for better responses it is necessary to employ various optimizing techniques to get the optimal cutting parameters and the theoretical models to do the predictions. Again optimization of multiresponse characteristics is more complex in comparison to single response. The multiresponse optimization principle is different from single response optimization. There is more than one objective function in multiresponse optimization, each of which may have a different solution [10, 11]. Işık and Kentli [12] proposed a multicriteria optimization approach for the responses such as maximization of material removal rate (MRR) and minimization of cutting forces which handles the possible manufacturing errors in design stage. The optimal result showed considerable performance characteristics improvement in machining process. Palanikumar et al. [13] used an orthogonal array and grey relational technique for converting the multiresponse to single response and optimizing the process parameters in GFRP composites with cemented carbide K-10 tool and got a considerable improvement in performance characteristics in machining process. Lin [14] used grey relational analysis for converting multiresponses such as tool life cutting force and surface roughness to a single response and showed clearly that the multiperformance characteristics in turning operations are greatly improved. Gupta and Kumar [15] used Grey relation analysis and principal component analysis for optimizing the process parameters and to find out the relative significance performance characteristics. The GRA and PCA conclude that depth of cut has great influence on surface roughness and MRR followed by feed rate. Nayak and Mahapatra [16] used AHP and TOPSIS method for optimization of multiresponses such as MRR, surface finish, and kerf and concluded that the methodology is capable of optimizing any type of problem with any number of responses. Gadakh [17] used TOPSIS method for solving multicriteria optimization problem in wire electrodischarge machining process. For optimal process parameter selection a good amount of research has been done using this area (TOPSIS) and most of the works used experimental data for the optimization.

In the present work, TOPSIS method has been applied to convert the multiresponses to an equivalent single response. Taguchi approach is used to analyze the effect of turning parameters such as speed, feed, and depth of cut. Optimization of process parameters for individual performance characteristics is found here and is verified by confirmation tests. Also statistical analysis of variance (ANOVA) is performed to judge the significance of factor for responses.

## 2. Methodology: TOPSIS

TOPSIS stands for technique for order preference by similarity to ideal solution. This method was developed by Hwang and Yoon in the year 1995. Technique for order preference by similarity to ideal solution (TOPSIS) is based on the idea that the chosen alternative should have the shortest distance from the positive ideal solution and on the other side the farthest distance of the negative ideal solution. The ideal solution is a hypothetical solution for which all attribute values correspond to the minimum attribute values in the data base. TOPSIS thus gives a solution that is not only closest to the hypothetically best but also farthest from the hypothetically worst. The steps followed for the TOPSIS in the present research work are given below.

*Step 1*. Decision matrix is normalized by using the following equation:

(1) $$\begin{array}{c} r_{ij}=\frac{a_{ij}}{\sqrt{\sum_{i=1}^{m}{a_{ij}}^{2\;}}}, \end{array}$$

where *i* = 1 ⋯ *m* and *j* = 1 ⋯ *n*. *a*_*ij*_ represents the actual value of the *i*th value of *j*th experimental run and *r*_*ij*_ represents the corresponding normalized value.

*Step 2.* Weight for each response is calculated.

*Step 3.* The weighted normalized decision matrix is then calculated by multiplying the normalized decision matrix by its associated weights. The weighted normalized decision matrix is formed as

(2) $$\begin{array}{c} V_{ij}=W_{i}\times r_{ij}, \end{array}$$

where *i* = 1 ⋯ *m* and *j* = 1 ⋯ *n*. *w*_*j*_ represents the weight of the *j*th attribute or criteria.

*Step 4.* Positive ideal solution (PIS) and negative ideal solution (NIS) are determined as follows:

(3) $$\begin{array}{c} V^{+}=\left({v_{1}}^{+},{v_{2}}^{+},\ldots ,{v_{n}}^{+}\right)\;\;\text{maximum}\;\;\text{values},\\ V^{-}=\left({v_{1}}^{-},{v_{2}}^{-},\ldots ,{v_{n}}^{-}\right)\;\;\text{minimum}\;\;\text{values}. \end{array}$$

*Step 5.* The separation of each alternative from positive ideal solution (PIS) and negative ideal solution (NIS) is calculated as

(4) $$\begin{array}{c} {S_{i}}^{+}=\sqrt{\overset{M}{\underset{j=1}{\sum }}{\left(v_{ij}-{v_{j}}^{+}\right)}^{2}},\\ {S_{i}}^{-}=\sqrt{\overset{M}{\underset{j=1}{\sum }}{\left(v_{ij}-{v_{j}}^{-}\right)}^{2}}\;\text{where}\;\;i=1,2,\ldots ,N. \end{array}$$

*Step 6.* The closeness coefficient of each alternative (CC_*i*_) is calculated as

(5) $$\begin{array}{c} \text{C}{\text{C}}_{i}=\frac{{S_{i}}^{-}}{{S_{i}}^{+}+{S_{i}}^{-}}. \end{array}$$

## 3. Experimental Work

The workpiece used for the present research is GFRP bar with 40 mm diameter. The GFRP bar is fabricated by dry hand lay-up technique. The hand lay-up technique is chosen as it is ideally suited to manufacture low volume with minimum tooling cost. The calculated amount of epoxy has been taken and as per the weight percentage of graphite powder is dispersed on an ultrasonic stirrer. The stirring is continued for one hour. The hardener is added to the epoxy graphite solutions and stirred manually for 10 minutes. Epoxy, graphite, and hardener solutions are spread over on an aluminium foil and the calculated amount of glass fiber placed layer by layer to make the GFRP composite. The mechanical properties of the composite material have been investigated and it is found that the tensile strength is 315.14 Mpa, tensile modulus is 6.2 Gpa, and the flexural strength is 343.43 Mpa. The experiments are planned using Taguchi's L_9_ orthogonal array that helps to reduce the number of experimental runs. The three cutting parameters such as speed, feed, and depth of cut with three different levels are used for the experimentation. The longitudinal turning experiments are carried out in an all geared lathe machine whose cutting speed ranges from 200 to 400 rpm and feed ranges from 0.03 to 0.05 mm/rev. The machining tests are carried out in a dry condition with carbide tooling having insert specification VNMG110408. During the experiments, single setup experiments have been carried out with different conditions of parameter setting. The composition of fiber, resin, and filler and the machining parameters with their levels are listed in Tables 1 and 2.

### 3.1. Material Removal Rate (MRR)

The material removal rate is the volume of material removed per unit time. Volume of material removed is a function of speed, feed, and depth of cut. Material removal with a higher rate is one of the most important criteria during the turning operation. The MRR is calculated using the expression

(6) $$\begin{array}{c} \text{MRR}=\pi D_{i}dfN\;\text{in}\;\;{\text{mm}}^{3}/\min, \end{array}$$

where *D*_*i*_ is the initial diameter, *d* is the depth of cut, *f* is the feed rate and *N* is the number of revolutions of spindle per minute.

### 3.2. Surface Roughness (*R*_*a*_)

Surface roughness is also another important aspect in machining of GFRP composite. Here the roughness is measured three times using a stylus type surface roughness tester (Taylor Hobson, Sutronic 25) of sampling length 0.8 mm, evaluation length of 4 mm, and least count of 0.01 *μ*m and the average roughness is listed in Table 2.

## 4. Optimization of Individual Performance Characteristics

### 4.1. Determination of Optimal Process Parameters for MRR

In this section, L_9_ orthogonal array is used to determine the effect of process parameters on individual characteristics using *S*/*N* ratio and ANOVA analysis. According to Taguchi method there are three performance characteristics such as higher-is-better, nominal-is-better, and lower-is-better. Here higher-is-better characteristics are used to find the optimal process parameter for MRR. The MRR and *S*/*N* ratio for MRR are shown in Table 3.

### 4.2. Analysis of *S*/*N* Ratio for MRR

As the experimental design is orthogonal, it is possible to separate out the effect of each process parameter at different levels. The term signal represents the desirable and the noise represents the undesirable and the response considering highest *S*/*N* ratio is close to optimal. The mean *S*/*N* ratio of material removal rate for cutting speed at levels 1, 2, and 3 can be calculated by averaging the *S*/*N* ratios for experiments 1–3, 4–6, and 7–9, respectively. Similarly the mean *S*/*N* ratio of material removal rate for other process parameters such feed and depth of cut can be computed. The mean *S*/*N* response table for material removal rate is shown in Table 4.

**Figure 1:**
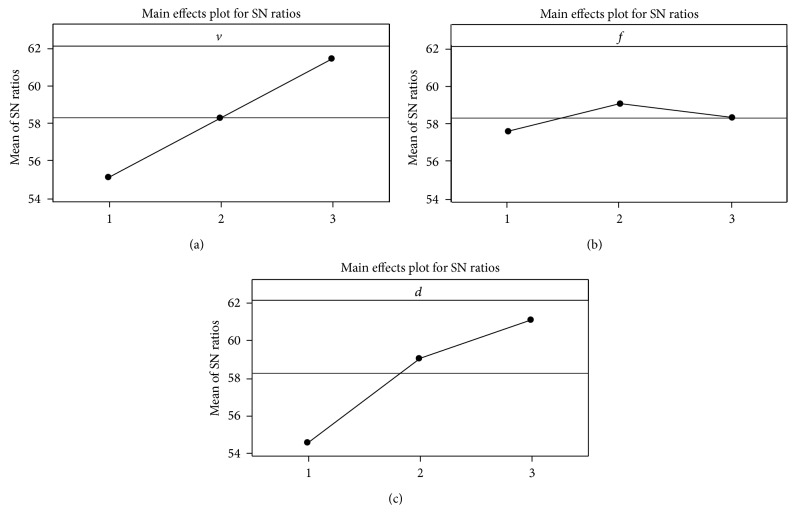
Mean *S*/*N* ratio graph for MRR. Signal-to-noise: larger is better.

The mean *S*/*N* ratio graph for material removal rate is shown in Figure 1. Optimal results can be found out from the main effects plot selecting the highest levels of *S*/*N* ratio values. Therefore, based on the *S*/*N* analysis, the optimal process parameters for material removal rate are cutting speed at level 3, feed at level 2, and depth of cut at level 3, that is, *v*_3_-*f*_2_-*d*_3_.

### 4.3. ANOVA for MRR

The purpose of the ANOVA is to find the statistical significance of process parameters for the response (MRR) shown in Table 5. This is accomplished by separating the total variability of the *S*/*N* ratios, which is measured by the sum of the squared deviations from the total mean of the *S*/*N* ratio, into contributions by each of the process parameters and the error [18]. *F*-test is performed to judge the significant parameter affecting the material removal rate. The larger *F*-value affects more the performance characteristics. From Table 5 it is found that cutting speed and depth of cut have *P* value less than 0.05, which means that cutting speed and depth of cut are significant at 95% confidence level. Also feed is to be found insignificant parameter for material removal rate from the ANOVA analysis. The *R* ^2^ value is high, close to 1, which is desirable.

### 4.4. Confirmation Test for MRR

Confirmation tests are carried out using the optimal process parameter combination to predict and verify the improvement of the performance characteristics as shown in Table 6. The estimated *S*/*N* ratio using the optimal combination of cutting parameters is calculated. The table shows the results of verification test and comparison between the actual machining performance and predicted machining performance. Good agreement exists between predicted and optimal value. Here the increase of *S*/*N* ratio from the initial process parameter to optimal process parameter is 12.8265 dB, and based on the confirmation test, MRR is increased 4.37 times.

### 4.5. Determination of Optimal Process Parameters for Surface Roughness (*R*_*a*_)

Similarly the *S*/*N* ratio for surface roughness is calculated. Here a smaller-is-better characteristic is used to find the optimal process parameter for surface roughness (*R*_*a*_). The *S*/*N* ratio for *R*_*a*_ is listed in Table 7. The mean *S*/*N* response table for surface roughness (*R*_*a*_) is shown in Table 8. It is clearly shown in the table that the rank of cutting speed is 1 which implies cutting speed is more significant for *R*_*a*_.

### 4.6. Analysis of *S*/*N* Ratio for Surface Roughness (*R*_*a*_)

**Figure 2:**
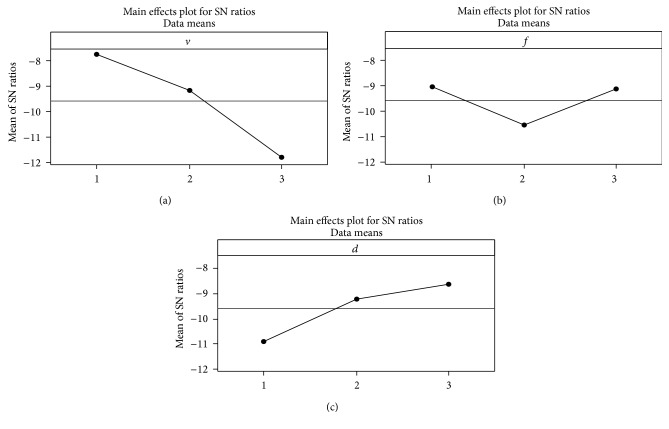
Mean *S*/*N* graph for surface roughness. Signal-to-noise: smaller is better.

Figure 2 shows the main effect on the surface roughness which is primarily due to cutting speed and depth of cut. The feed is found to be insignificant from the main effects plot. Therefore, based on the *S*/*N* analysis and main effects plot for *S*/*N* ratio, the optimal process parameters for surface roughness are cutting speed at level 1, feed at level 1, and depth of cut at level 3, that is, *v*_1_-*f*_1_-*d*_3_.

### 4.7. ANOVA for Surface Roughness (*R*_*a*_)

ANOVA for surface roughness (*R*_*a*_) is listed in Table 9. From the table it is clearly found that speed with a *P* value less than 0.05 means it is significant at 95% confidence level. So, speed is the most significant parameter for *R*_*a*_ followed by depth of cut and feed. The *R* ^2^ value is also high, close to 1, which is desirable.

### 4.8. Confirmation Test for Surface Roughness (*R*_*a*_)

In the confirmation table for surface roughness (*R*_*a*_), it is found that the increase of *S*/*N* ratio from the initial process parameters to the optimal process parameter is 2.7244 dB, which is shown in Table 10. Based on the confirmation test it is also found that the surface roughness is increased 1.36 times.

## 5. Simultaneous Optimization Using TOPSIS Method

Initially the two responses such as *R*_*a*_ and MRR are normalized using (1), which is shown in Table 11. In the present work as same priority is given to both surface roughness as well as to MRR, so for both responses weight criterion is taken as 0.5; that is, *W*_*Ra*_ = 0.5 and *W*_MRR_ = 0.5. With the proper weight criteria the relative normalized weight matrix has been calculated. The weight criteria are multiplied to get the normalized weighted matrix using (2) as shown in Table 12. The ideal and the negative ideal solutions are calculated from the normalized weighted matrix table; that is, *V*_*Ra*_^+^ = 0.104094126, *V*_*Ra*_^−^ = 0.253989668 and *V*_MRR_^+^ = 0.243138323, *V*_MRR_^−^ = 0.055767928. The separation measures of each criterion from the ideal and negative ideal solutions are calculated using (4), which is shown in Table 13. Finally the relative closeness coefficient (CC_*i*_) value for each combination of factors of turning process is calculated using (5), which is shown in Table 14.

**Figure 3:**
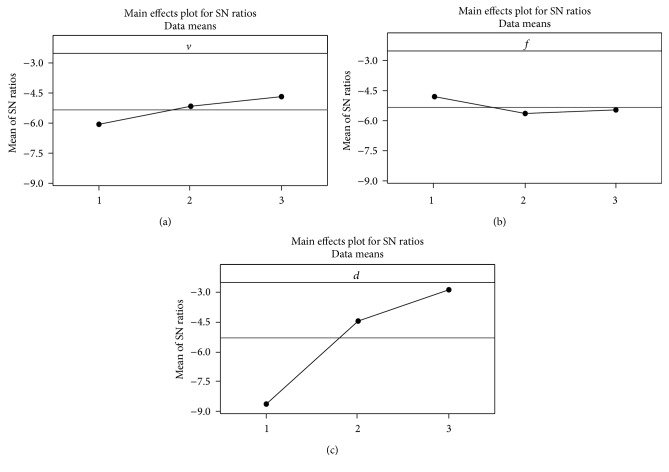
Response plot for closeness coefficient CC_*i*_.

**Figure 4:**
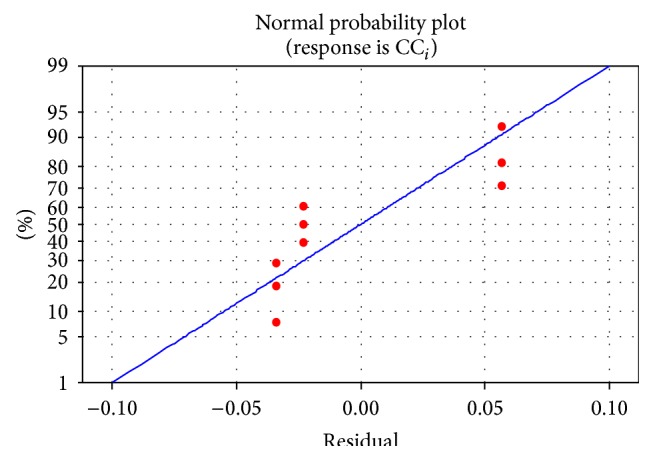
Normal probability plot for CC_*i*_.

The response table of mean *S*/*N* ratio for closeness coefficient value is shown in Table 15. The effect of depth of cut for the combine response is most significant. The optimal machining parameters obtained from the response graph are shown in Figure 3. The optimal input parameters for the GFRP machining are cutting speed at level 3, feed at level 1, and depth of cut at level 3 for minimizing the surface roughness and maximizing the material removal rate. The analysis of variance and the percentage contribution of each parameter are shown in Table 16. It shows that the contribution of depth of cut is more enough for combined response. From the normal probability plot of residuals in Figure 4, it is evident that the residuals lie reasonably close to a straight line implying that errors are distributed normally. This gives the support that the terms mentioned in the model are significant.

After the identification of optimum level of machining parameters the final step is to predict and verify the improvement of performance characteristics using the optimal level of machining parameters.  Table 17 shows the comparison of the estimated closeness coefficient value with the actual closeness coefficient value obtained in experiment using the optimal cutting parameters. Good agreement between the predicted machining performance and actual machining performance is shown in Table 17. The increase of the closeness coefficient value from the initial cutting parameters to the optimal cutting parameter is 0.322.

## 6. Conclusion

Though a lot of methods for multiresponse optimization in machining processes are studied, a new method (TOPSIS) is proposed in this work. The main advantage of this method is that there is no need to calculate complex modeling formulations or simulations of process, which takes a lot of time and hardware to find out the optimum solution. Instead of the complex modeling formulations, a simple statistical calculation has been used to get the appreciable result. This approach also gives much more reliable solutions as exact experimental values are used to represent the process. When the results are compared it is found that depth of cut is a factor which has great influence on increasing MRR and decreasing *R*_*a*_ followed by speed and feed. It is also observed that there is a good agreement between the estimated value (0.782267) and the experimented value (0.71686) using TOPSIS method for multiresponse optimization. The improvement of closeness coefficient (CC_*i*_) from initial parameter combination (*v*_2_-*f*_2_-*d*_1_) to optimal parameter combination (*v*_3_-*f*_1_-*d*_3_) is 0.322. The experimental result for optimum setting shows that there is a considerable improvement in the performance characteristics. Thus the multiresponse approach using TOPSIS with Taguchi approach is capable of solving any type of optimization problem.

## Figures and Tables

**Table 1:** Specification of epoxy, filler, and fiber.

Sl. number	Epoxy + hardener	Filler	E-glass
01	Araldite LY 556 + HY 951 (50 wt%)	Graphite (3 wt%) + ash clay (3 wt%)	R099 1200 P566 (50 wt%)

**Table 2:** L_9_ orthogonal array with experimental data.

Expt. number	*v* (rpm)	*f* (mm/rev)	*d* (mm)	*v* (actual)	*f* (actual)	*d* (actual)	MRR in (mm^3^/min)	Surface roughness *R*_*a*_ in (*µ*m)
1	1	1	1	200	0.03	0.5	319.49	2.60
2	1	2	2	200	0.04	1.0	625.8	2.80
3	1	3	3	200	0.05	1.5	913.26	2.00
4	2	1	2	300	0.03	1.0	971.28	2.50
5	2	2	3	300	0.04	1.5	1155.08	2.80
6	2	3	1	300	0.05	0.5	493.13	3.40
7	3	1	3	400	0.03	1.5	1392.92	3.52
8	3	2	1	400	0.04	0.5	974.42	4.88
9	3	3	2	400	0.05	1.0	1211.15	3.44

**Table 3:** Experimental results for MRR and *S*/*N* ratio.

Run	*v*	*f*	*d*	MRR (mm^3^/min)	*S*/*N* ratio
01	1	1	1	319.49	50.0891
02	1	2	2	625.8	55.9287
03	1	3	3	913.26	59.2119
04	2	1	2	971.28	59.7469
05	2	2	3	1155.08	61.2522
06	2	3	1	493.13	53.8592
07	3	1	3	1392.92	62.8785
08	3	2	1	974.42	59.7749
09	3	3	2	1211.15	61.6640

**Table 4:** Response table of mean *S*/*N* ratio for MRR.

Symbol	Process parameters	Mean *S*/*N* ratio				Rank
		Level 1	Level 2	Level 3	Max.−min.	
*v*	Speed	55.08	58.29	61.44	6.36	2
*f*	Feed	57.57	58.99	58.25	1.41	3
*d*	Depth of cut	54.57	59.11	61.11	6.54	1
Total mean *S*/*N* ratio = 58.26 dB						

**Table 5:** ANOVA table for MRR.

Source	DF	SS	MS	*F*	*P*
*v*	2	495212	247606	24.66	0.039
*f*	2	3165	1582	0.16	0.864
*d*	2	474699	237349	23.64	0.041
Error	2	20081	10040		

Total	8	993156			

*S* = 100.202; *R*-Sq = 97.98%; *R*-Sq (adj) = 91.91%.

**Table 6:** Results of confirmation for MRR.

	Initial process parameter	Optimal process parameter	
		Prediction	Experiment
Level	*v*_1_-*f*_1_-*d*_1_	*v*_3_-*f*_2_-*d*_3_	*v*_3_-*f*_2_-*d*_3_
MRR	319.49		1398.88
*S*/*N* ratio (dB)	50.0891	65.0041	62.9156
Improvement of *S*/*N* ratio = 12.8265 dB			

**Table 7:** Experimental results for surface roughness and *S*/*N* ratio.

Run	*v* (rpm)	*f* (mm/rev)	*d* (mm)	*R*_*a*_ (*µ*m)	*S*/*N* ratio
01	1	1	1	2.60	−8.2995
02	1	2	2	2.80	−8.9432
03	1	3	3	2.00	−6.0206
04	2	1	2	2.50	−7.9588
05	2	2	3	2.80	−8.9432
06	2	3	1	3.40	−10.6296
07	3	1	3	3.52	−10.9309
08	3	2	1	4.88	−13.7684
09	3	3	2	3.44	−10.7312

**Table 8:** Response table of mean *S*/*N* ratio for *R*_*a*_.

Source	Process parameters	Mean *S*/*N* ratio				Rank
		Level 1	Level 2	Level 3	Max.−min.	
v	Speed	−7.754	−9.177	−11.810	4.056	1
f	Feed	−9.063	−10.552	−9.127	1.489	3
d	Depth of cut	−10.899	−9.211	−8.632	2.268	2
Total mean *S*/*N* ratio = −9.580 dB						

**Table 9:** ANOVA table for surface roughness (*R*_*a*_).

Source	DF	SS	MS	*F*	*P*
*v*	2	3.47369	1.73684	26.46	0.036
*f*	2	0.68862	0.34431	5.25	0.160
*d*	2	1.25662	0.62831	9.57	0.095
Error	2	0.13129	0.06564		

Total	8	5.55022			

*S* = 0.256212; *R*-Sq = 97.63%; *R*-Sq (adj) = 90.54%.

**Table 10:** Results of confirmation for surface roughness (*R*_*a*_).

	Initial process parameter	Optimal process parameters	
		Prediction	Experiment
Level	*v*_1_-*f*_1_-*d*_1_		*v*_1_-*f*_1_-*d*_3_
*R*_*a*_	2.60		1.9
*S*/*N* ratio (dB)	−8.2995	−6.28783	−5.5751
Improvement of *S*/*N* ratio = 2.7244 dB			

**Table 11:** Normalization of the responses.

Sl. number	*R*_*a*_	MRR
1	0.270644728	0.111535857
2	0.291463553	0.218470497
3	0.208188252	0.318824490
4	0.260235315	0.339079617
5	0.291463553	0.403245289
6	0.353920029	0.172154612
7	0.366411324	0.486276646
8	0.507979335	0.340175810
9	0.358083794	0.422819659

**Table 12:** Normalized weighted matrix.

Sl. number	*R*_*a*_	MRR
1	0.135322364	0.055767928
2	0.145731777	0.109235249
3	0.104094126	0.159412245
4	0.130117658	0.169539809
5	0.145731777	0.201622644
6	0.176960014	0.086077306
7	0.183205662	0.243138323
8	0.253989668	0.170087905
9	0.179041897	0.211409829

**Table 13:** Separation measures.

Sl. number	S_i_^+^	S_i_^−^
1	0.189955	0.11867
2	0.140227	0.12074
3	0.083726	0.18224
4	0.078064	0.16819
5	0.058798	0.18164
6	0.173140	0.08278
7	0.079112	0.20029
8	0.166748	0.11432
9	0.081387	0.17275

**Table 14:** Closeness coefficient value.

Sl. number	CC_*i*_	*S*/*N* ratio
1	0.38451	−8.30185
2	0.46267	−6.69457
3	0.6852	−3.28365
4	0.683	−3.31159
5	0.75545	−2.43589
6	0.32346	−9.80359
7	0.71686	−2.89131
8	0.40673	−7.81388
9	0.67975	−3.35302

**Table 15:** Response table of mean *S*/*N* ratio for CC_*i*_.

Symbol	Process parameters	Mean *S*/*N* ratio				Rank
		Level 1	Level 2	Level 3	Max.−min.	
*v*	Speed	−6.093	−5.184	−4.686	1.407	2
*f*	Feed	−4.835	−5.648	−5.480	0.813	3
*d*	Depth of cut	−8.640	−4.453	−2.870	5.769	1
Total mean *S*/*N* ratio = −5.321 dB						

**Table 16:** ANOVA table.

Source	DF	Seq SS	Adj SS	Adj MS	% contribution
*v*	2	0.014202	0.014202	0.007101	6.24%
*f*	2	0.004299	0.004299	0.002150	1.89%
*d*	2	0.189207	0.189207	0.094603	83.24%
Error	2	0.019585	0.019585	0.009793	

Total	8	0.227294			

**Table 17:** Results for the confirmation experiment.

	Initial machining parameters	Optimal cutting parameters	
		Prediction	Experiment
Level	*v*_2_-*f*_2_-*d*_1_	*v*_3_-*f*_1_-*d*_3_	*v*_3_-*f*_1_-*d*_3_
*R*_*a*_	4.38		3.52
MRR	992.92		1392.92
Closeness coefficient value	0.39462	0.782267	0.71686

Improvement of closeness coefficient value = 0.322.
